# Transitioning from Forensic Genetics to Forensic Genomics

**DOI:** 10.3390/genes9010003

**Published:** 2017-12-22

**Authors:** Manfred Kayser, Walther Parson

**Affiliations:** 1 Department of Genetic Identification, Erasmus MC University Medical Center Rotterdam, 3000 CA Rotterdam, The Netherlands; 2 Institute of Legal Medicine, Medical University Innsbruck, 6020 Innsbruck, Austria; 3 Forensic Science Program, The Pennsylvania State University, University Park, PA 16802, USA

## Abstract

Due to its support of law enforcement, forensics is a conservative field; nevertheless, driven by scientific and technological progress, forensic genetics is slowly transitioning into forensic genomics. With this Special Issue of *Genes* we acknowledge and appreciate this rather recent development by not only introducing the field of forensics to the wider community of geneticists, but we do so by emphasizing on different topics of forensic relevance where genomic, transcriptomic, and epigenomic principles, methods, and datasets of humans and beyond are beginning to be used to answer forensic questions.

Although being a conservative field per se because of serving law enforcement, forensic genetics is slowly transitioning into forensic genomics. With this Special Issue of *Genes* we acknowledge and appreciate this rather recent development. Genomic, transcriptomic, and epigenomic principles, data, and technologies are applied to identify and analyze useful DNA and RNA markers to address various forensic questions that cannot be answered, or only in a limited way, via genetic or other approaches. Human genome data produced with SNP microarray technologies, and increasingly whole exome and whole genome data established via massively parallel sequencing (MPS) technologies, are used to identify DNA markers for individual identification as well as for appearance and ancestry prediction. The latter is forensically relevant for finding unknown perpetrators of crime who are unidentifiable with standard DNA profiling. Human transcriptome data of various tissues generated with expression microarray technologies, and increasingly with whole transcriptome sequencing via MPS technologies, are used to identify RNA markers to determine the cellular source of crime scene sample. This is forensically relevant for reconstructing the course of events that may have happened at the scene of crime and to support the use of DNA at the activity level of evidence interpretation. Human epigenome data established with DNA methylation microarray technologies, and increasingly by whole epigenome sequencing via MPS technologies, are used to identify DNA methylation markers for forensic tissue identification, for predicting lifetime age suitable for finding unknown perpetrators and for differentiating identical twins, the latter two cannot be identified via standard DNA profiling. Targeted MPS technologies, partly combined with hybridization capture and primer extension capture technologies, are applied to analyze forensic DNA and RNA markers with increased throughput, which improves various forensic applications such as individual and lineage identification, appearance, and ancestry prediction, as well as the interpretation of DNA mixtures produced from more than one person. Non-human genomic and transcriptomic data such as those from insects or microbes are useful in the forensic context—e.g., to estimate time of death—and for microbial species and strain identification to solve cases of bioterrorism. Several of these developments are covered by the articles collated in this Special Issue that we have the privilege to serve as guest editors for. Thus, we not only aim to introduce the field of forensics to the wider community of geneticists, but also to do so by emphasizing topics where genomic principles, methods, and datasets are beginning to be employed in the forensic context.

## Brief Guest Editors’ Biographies

Prof. **Walther Parson** holds an associate professorship at the Institute of Legal Medicine, Medical University of Innsbruck, Austria and an adjunct professorship at PennState, PA, USA. Under his scientific supervision the Austrian National DNA Database Laboratory was set up in Innsbruck where he is currently overseeing the High Through-put DNA Database Laboratory and the research group on Forensic Genomics. He is representing Austria on international boards including the European Network of Forensic Science Institutes (ENFSI) DNA Working Group or the European DNA Profiling Group (EDNAP) and he is an elected active member of the Academy of Sciences Leopoldina. He current serves as President of the International Society for Forensic Genetics (ISFG). He was repeatedly consigned to handle international requests on DNA fingerprinting of victims of mass fatalities (e.g., 2004 Tsunami, 1973 Chile, 2014 Mexican students), international human identification cases (e.g., Birgitta Tengs Case, Isdalen woman) and historic individuals (Russian Tsar family Romanov, Friedrich von Schiller, Wolfgang Amadeus Mozart, Günter Messner, Dark Countess, Leopold III). His research group is interested in various fields of genetics and genomics, including forensics, medical and population genetics and entertains collaboration with other fields of research such as anthropology, archaeology, mathematics and history. Walther Parson and his group have developed and maintain the EDNAP Mitochondrial DNA Population Database (EMPOP), the world’s largest forensic mitochondrial DNA database for forensic quality control and data interpretation purposes. He and his group recently developed the STRs for Identity ENFSI Reference Database (STRidER) to quality control and disseminate STR allele frequencies and sequenced alleles. He has published more than 300 peer-reviewed original articles.

Prof. **Manfred Kayser** is (full) Professor of Forensic Molecular Biology at Erasmus University Rotterdam and heads the Department of Genetic Identification at Erasmus University Medical Center (2004-today). He received his diploma in biology from University of Leipzig (1994), Ph.D. in biology/genetics with *summa cum laude* from Humboldt University Berlin (1998), and habilitation in genetics from University of Leipzig (2004). After postdoctoral research at Pennsylvania State University, Department of Anthropology, he was staff scientist and later Heisenberg Fellow of the German Research Council at Max Planck Institute for Evolutionary Anthropology Leipzig, Department of Evolutionary Genetics (1999–2004). His research interest is in various aspects of human, forensic, and anthropological genetics. In the forensic world, he is well known for the introduction and further development of forensic Y-chromosome analysis and Forensic DNA Phenotyping, and additionally published on molecular tissue identification, molecular time estimation, molecular age estimation, genetic ancestry inference and molecular and functional genetics of human appearance. As of 2017, he (co)authored 215 peer-reviewed articles in scientific journals (H factor > 55). He serves/d as editor-in-chief, guest editor, academic editor, editorial board and advisory board member of several forensic and genetics journals and is regular ad-hoc reviewer for various scientific journals and granting agencies. He received the Scientific Price 1998 of the German Society of Legal Medicine and the Biennial Scientific Price 2017 of the International Society for Forensic Genetics.

